# Toward a digital citizen lab for capturing data about alternative ways of self-managing chronic pain: An attitudinal user study

**DOI:** 10.3389/fresc.2022.942822

**Published:** 2022-08-15

**Authors:** Najmeh Khalili-Mahani, Sandra Woods, Eileen Mary Holowka, Amber Pahayahay, Mathieu Roy

**Affiliations:** ^1^McGill Centre for Integrative Neuroscience, Montreal Neurological Institute, Montreal, QC, Canada; ^2^Department of Design and Computation Arts, Faculty of Fine Arts, Concordia University, Montreal, QC, Canada; ^3^PERFORM Centre, Concordia University, Montreal, QC, Canada; ^4^Quebec Pain Research Network (QPRN), Sherbrooke, QC, Canada; ^5^Patient Partner, Montreal, QC, Canada; ^6^Department of Communication Studies, Faculty of Arts and Science, Concordia University, Montreal, QC, Canada; ^7^School of Public Health and Health Systems, University of Waterloo, Waterloo, ON, Canada; ^8^Department of Psychology, Faculty of Science, McGill University, Montreal, QC, Canada

**Keywords:** chronic pain, data sharing, citizen science, attitudinal survey study, non-pharmacological pain treatment, mHealth, mixed-methods research, patient-oriented research

## Abstract

**Background:**

Myriad psychosocial and cultural factors influence personal ways of coping with chronic pain (CP). Mobile health (mHealth) apps facilitate creation of citizen laboratories outside clinical frameworks. However, issues of safety, privacy and technostress must be addressed. This attitudinal user study aimed to assess whether persons with persistent pain (PwPP) would be open to sharing qualitative and quantitative data about their self-management of CP *via* mHealth platforms.

**Methods:**

In March 2020, we invited PwPPs, their personal or medical caregivers, or those interested in the development of an app for researching alternative ways of self-managing CP to complete an anonymous survey. We formulated an attitudinal survey within the theoretical framework of stress to estimate whether the novelty, unpredictability, and risks of data-sharing *via* mHealth apps concerned users. Descriptive statistics (% Part/Group) were used to interpret the survey, and open comments were reflectively analyzed to identify emerging themes.

**Results:**

Of 202 responses (June 2021), 127 identified as PwPPs (average age 43.86 ± 14.97; 100/127 female), and listed several primary and secondary CP diagnoses. In almost 90% of PwPPs, physical and emotional wellbeing were affected by CP. More than 90% of PwPPs used alternative therapies (acupuncture, homeopathy, massage therapy, etc.). Attitude toward mHealth apps were positive even though nearly half of PwPPs were unfamiliar with them. More than 72% of respondents were *open to using a health-related app as a research tool for data collection in real life situations*. Comprehensive data collection (especially about psychosocial factors) was the most important requirement. More respondents (especially medical professionals) were concerned about health hazards of misinformation communicated *via* health-related information and communication systems (maximum 80%) than about privacy (maximum 40%). Qualitative analyses revealed several promises and impediments to creation of data-sharing platforms for CP.

**Conclusions:**

This study shows a general willingness among PwPPs to become partners in studying alternative pain management. Despite a generally positive attitude toward the concept of sharing complex personal data to advance research, heterogeneity of attitudes shaped by personal experiences must be considered. Our study underlines the need for any digital strategy for CP research to be person-centered and flexible.

## Introduction

Chronic pain (CP), experienced by at least 25% of the population in North America, continues to stymie biomedical models and pharmacological interventions (1, 2). In Canada (with a universal healthcare system), where this study took place, nearly 8 million people live with CP. Aligned with the World Health Organization, Canada recognizes CP as a disease, in and of itself, and diagnoses it as a condition in which “*pain continues for longer than 3 months, with no known cause; after injury has healed; and after the condition has been treated*.” Conditions such as fibromyalgia, chronic pelvic pain, chronic musculoskeletal pain, and non-specific lower back pain are considered chronic primary pain, and post-surgical pain, rheumatoid arthritis or pain associated with cancer are considered chronic secondary pain (3).

The Canadian Pain Task Force report: June 2019 (4) emphasized the need for a Strategy for Patient-Oriented Research (SPOR), suggesting that “people living with chronic pain must be equal partners in research.” Key action elements included viewing and managing pain as a public health issue with confounding biopsychosocial factors, in need of globally coordinated action; identifying efficient and effective assessment tools to inform correct pathways of care; person-centered and flexible care models reflective of individual needs and experiences; accessible pain education for the public and professionals, to minimize the risks of stigma and implicit biases; and finally, the creation of national data collection methodologies to reliably evaluate different types of interventions (pharmacological, physical, psychological and alternative).

Two years later, this Task Force's March 2021 report (5) called pain a national emergency. As in previous years, it proposed an action plan for equitable and collaborative action at political, medical, and community levels to address the social, psychological, and economic burdens of this debilitating health condition. Adding to the 2019 emphasis on patient-partnership in research, the new strategic research priorities shifted towards technology, in order to “*support research efforts to improve processes, technology and interventions in the area of digital technologies for health including e-health and virtual care.”* One aim of accelerating research was said to be implementing a surveillance system to “*explore the potential of big data approaches that incorporate different data types from novel sources.”*

Research that we present in this article is part of a larger project to develop a health-related information and communication (ICT) platform aiming to involve PwPPs in studying two important issues: first, the influence of cultural, psychosocial, and environmental context in which one experiences pain; second, a knowledgebase from individuals' personal experiences of coping with CP outside clinical frameworks (6). Throughout this paper, “alternative” refers to any methods that are not within the mainstream medical practice and not covered by standard insurance plans. We have conceptualized this framework as a potential digital citizen laboratory for coping with untreatable persistent pain through creative and self-expressive ways.

## Background

A 2016 meta-analytical review of Citizen Science literature identified ecology, geography, and epidemiology as the three fields to benefit from massive data-collection (7). There is growing evidence to suggest that digital citizen laboratories for pain research are also likely to succeed. In a recent scoping review of more than 1200 clinical trials, we found a significant number of digital interventions used to conduct experimental quantitative pain research (8). In *Coping with Illness Digitally*, Stephan Rains illustrates the significance of different types of internet-based communication systems such as weblogs, and social networks in reinforcing connections; soliciting and providing social support; sharing experiences and seeking information; and even improving patient-provider relationships (9). Computerized clinical decision support systems have been long introduced (10), and applied in chronic pain management (11). With access to personal computers and the internet, it has become possible for pain patients to record and report the contextual variations in their daily experiences of pain digitally (12, 13); and to generate data about self-management techniques outside of clinical settings (14). Finally, it is expected that the accumulation of self-tracking data will make digital phenotyping easier (12, 15, 16). Data portals such as *PatientsLikeMe* aim to help individuals “*Find support from real people just like you and start taking charge of your health”*. As of April 2022, this portal enables 850,000 patients to conduct *N* = 1 self-trials (17), by empowering them to “*[...]compare treatments, symptoms & medication side effects. Track & monitor [their] own personal health data in real-time [...]*” while also creating a body of knowledge by exchanging personal experiences about symptoms and medications (18).

Mobile health applications (mHealth apps) have been widely researched and developed to facilitate personal pain management, and communication with healthcare providers. In a 2015 review “*There's an App for That Pain*,” Lalloo listed nearly 300 pain apps for personal and research purposes (19) [a count that has reached 508 market-place apps by the end of 2021 (20)]. More recent apps such as *Manage My Pain* include features that help patients track their pain, function, and medication; respond to questionnaires; and make those reports and data available to clinicians who can remotely study clinically relevant trends and discover patterns using advanced analytics (21). While it was shown that *Manage My Pain* was effective in reducing anxiety and pain in the short term, the authors reported a need for improving the conditions to sustain user engagement. In fact, including users in designing mHealth apps for pain is often mentioned as the most important factor to make them useful in clinical practice and research (22).

In the context of the 2019 Task Force priorities, we proposed a Digital Strategy for Play Oriented Research and Action (DiSPORA) (6). In conditions as complex as chronic pain, where clinical interventions have not provided satisfactory relief, those affected by CP may try (*play* with) alternative choices for which sufficient clinical evidence is not available. Therefore, DiSPORA aimed to serve as a digital citizen laboratory to facilitate gathering large-scale qualitative data about self-experimentation and self-reporting of information that *users* deemed to be important as they played with various self-care options (23). This conceptual framework recognizes the need for adopting person-centered approaches to pain management and research (24). Given that alternative therapeutics are not accessible through or recommended by the standard healthcare systems, we aimed to develop a digital citizen lab to report on how one's experiences with non-pharmacological pain management impacts them.

Citizen science can be viewed as participatory action research that aims to democratize (25) and personalize healthcare research (26, 27). This requires participants to contribute knowledge from their lived experiences and contextual actions (28, 29). However, in a digital citizen lab, adding a layer of technology and algorithmic opacity to how data is generated, shared, and interpreted, may challenge the reciprocity and balance of power that is expected from participants. For this reason, user participation and acceptance is vital in the earliest steps of technology development (30).

### Aims of the current study

This study aimed to assess whether the idea of an mHealth App to generate data from personal experiences, for advancing qualitative and participatory pain research, might be stressful to potential users. When technologies are first introduced, they cause what Brod (31) coined “technostress.” Technostress results from creating functional or emotional overload, ambiguity about its benefits, physical or financial inaccessibility, and potential for invasiveness in one's life. It is plausible that introducing a technology that resembles a “public health surveillance system” for data generation would raise concerns about safety, privacy, and even equitable access to it.

The most obvious technostress (exacerbated by the digitization of healthcare since the onset of the COVID-19 pandemic) would be “surveillance creep;” i.e., when data collected for a specific purpose (e.g., traffic control) is later exploited for another use (e.g., facial recognition) (32). Another common source of technostress is caused by pressuring users to invest time and resources to learn and adopt them into their lives. A recent study in three Swiss psychiatric hospitals indicated that the introduction of digital technologies among healthcare professionals is causing them technostress (33).

The concept of studying pain in a qualitative and 'creative' framework that centers around communicating data on alternative ways of coping with pain might be just as stressful as the new technology itself. There has been a traditional tendency to dismiss alternative medicine as “quack science” (34, 35), despite the fact that the long-term efficacy of many pharmacological treatments for chronic pain is also debated (36–38). This bias seems to be stronger in physicians who are dismissive of the medically unexplained pain of their patients (39, 40). This attitude is gradually changing. According to a World Health Organization report, in 2019, 170 member states had acknowledged using complementary alternative medicine (41). Although the practice has been on the rise in the European and American nations, the adoption is reported to be markedly slower than in other nations.

Our motivation for creating a play-oriented digital citizen lab derives in part from these kinds of cultural and other biases that fail to account for the personal narratives of those who resort to non-pharmacological treatments. Accessibility and empowerment of patients can help overcome technostress and predict effective uptake of such technologies for mental e-health purposes (42). Within our proposed framework, we hope to foster a more democratic and participatory engagement with the psychosocial and cultural complexity of how pain is communicated and cared for. Thus, capturing the attitudes of target users of digital health interventions is an essential first step in addressing concerns about their effectiveness (43, 44).

### Theoretical framework

In providing a perspective on the Law of Attrition (45), Eysenbach recommended that researchers develop a scientific framework to explain the reasons why a large proportion of eHealth solutions suffer high rates of drop-outs, discontinuation of use, or non-adoption. The challenge of attrition is also reported in mHealth apps for pain (22, 46, 47).

A suitable ecological and flexible framework for addressing this question is the Lazarus and Folkman's Transactional Theory of Stress and Coping (TTSC), which posits that when individuals are confronted with a novel experience, they recursively evaluate its relevance, beneficence, and risks against their existing resources, and would engage with or react to it based on their perceptual and adaptive strategies (e.g., cognitive or emotional, or avoidance and approach) (48). The Transactional Theory of Stress has been applied to the question of technostress when new digital technologies are introduced into the workplace (49–51) or, conversely, to examine whether ICTs can help alleviate stress (52–54).

Briefly, this model suggests that their primary appraisal (attitudes) towards the benefits or threats of a new challenge will determine whether a user would choose to approach or avoid it when it is first presented. In our case, those who have a strong negative attitude about the new technology are unlikely to participate in its development and testing. But, if there is an interest to consider the potential benefits, those who are ambivalent might try it and go on to develop a new attitude over time as they recursively examine the efforts needed to achieve gains (secondary appraisal). In this appraisal process, benefits are re-evaluated against the actual costs (psychological or material) of adopting the technology into their lives. Novelty, unpredictability, threat to self and sense of control (N.U.T.S) are predictors of stressful responses to new conditions (47, 48), particularly for chronic pain patients (55). If PwPPs do not have control over the use or refusal of a new technology that is introduced into their care, and this increases their uncertainty and threatens their self-care, they will experience 'technostress' (31, 56) and likely discontinue its use. What makes TTSC suitable for health related ICT design studies is its sensitivity to stress (as a psychobiological phenomenon)—an adaptive and dynamic process that is recursively informed by the active learning and decision-making of their users (primary and secondary appraisal) against the resources that they possess (physical, psychological and cognitive state). TTSC provides an empirical framework that accounts for (and allows experimental manipulation of) myriad factors that moderate one's physiological, psychological, and physical interactions with stressors (in our case, a chronic illness) and de-stressors (in our case, a self-research tool intended to empower and inform patients), which can be quantitatively measured (e.g., from stress hormones, electrophysiological brain responses, or autonomic responses). We have elsewhere elaborated on how this theoretical framework can be utilized to develop assistive ICTs (57), and have been testing the model in various studies in the relationship between ICT use and stress relief (58–60).

## Methods

### Study design

The current study sought to investigate the attitudes of potential stakeholders towards creation of a digital citizen laboratory for chronic pain. Our attitudinal methods aimed to develop an understanding of the overarching needs, beliefs, and general motivations of targeted users. Using simple but broad questions, we solicited opinions of would-be-users on the perceived appeal, quality, and/or usefulness of a design or any of its individual elements (61). As such, we formulated a short survey to capture the potential sources of N.U.T.S in our proposal by evaluating the degree of familiarity and general attitude of targeted stakeholders towards mHealth apps, in general, and personal data-sharing, in particular.

### Sampling

With institutional ethics (REB) approval, we invited individuals (patients, caregivers, or healthcare professionals) who were *interested in helping to design an app for studying the creative ways of coping with pain* to join our study. A link to the study website offered explanations about the objective of the app, “*A Citizen's Laboratory for researching non-pharmacological treatments for chronic pain*,” and invited participants to join our team efforts:

“*We all have experienced pain, but our experiences are unique. We have unique ways of coping with pain too. Sometimes medications or physical therapies don't work. Sometimes meditation or cognitive therapies do work. Some of us might play. Some of us might pray. We want our unique ways of coping to be considered in research and care. This will guarantee that our healthcare systems will be inclusive and respectful of our specific needs. To become partners in research will help scientists design more targeted systems for personalized care or cure.”*

The first step to join this partnership was an anonymous online survey (SurveyMonkey).

The invitation and link to the survey were sent to members of a mailing list of research participants of Concordia University's PERFORM Centre, disseminated *via* the social media of researchers (Twitter, Facebook). The same link was shared across both the PwPP and bioethics/healthcare networks of our Patient Partner co-author (pain research groups, chronic pain patient associations, Facebook, Instagram, Twitter, her blog, and in conversations). Paper pamphlets were also distributed throughout the PERFORM Centre's athletic therapy unit. The survey was anonymous. Participants who wished to be involved in the next stage of the design were not identifiable from this survey, which was offered in both English and French (the official languages of Canada).

### Survey questions

In general, we were interested in learning about the extent of familiarity of our target users with mHealth applications (Novelty), and their attitudes towards these in terms of projected benefits and risks associated with their use as a data-generation tool (Unpredictability, Threat to self, and Sense of Control).

We did not intend this to be a quantitative psychometric study, rather aimed to conduct a quasi-qualitative opinion survey by asking questions in a colloquial and conversational manner. We avoided jargon, acronyms, and other uncommon terms (such as eHealth, mHealth, Citizen Lab), and provided the flexibility to skip questions, to select more than one option (e.g., for type of pain, or reasons for participation), and allowed variable answer options (such as 'Maybe' or 'I don't know') in order to capture any ambivalence in responses, and to invite additional comments.

The categories of information for which we screened included:

- Reason for interest in the project (I have persistent pain; I am a caregiver to someone with persistent pain; I am a medical professional; I am a digital-health designer and researcher; I am a policy-maker; I am just curious).
- Demographics: age, gender, income, education, employment.
- Types of pain experienced by the PwPP, and the Impact of CP on different aspects of life (mobility, creativity, sociability, emotional wellbeing, hobbies, work, exercise).
- Current methods of coping with pain (medications, various therapies, distractions, activities), and interest in trying new techniques (mindfulness, hypnosis, virtual reality, different art therapies).
- Attitude towards alternative medicine (effectiveness, promise, safety).
- Access to mobile ICTs and usage.
- Attitude towards various forms of ICT (blogs, YouTube, official channels, social networks, apps, etc.) with regards to safety, privacy, and accuracy of health-related information exchange.
- Attitude towards mHealth applications, and their promise – if any—for research.
- Attitude towards data sharing (data types and data-collection system features).

In addition, we solicited comments on the following topics:

- Is there anything that you think would improve a pain-tracking app?
- To what extent do you agree with the following statements about pain evaluation:

- “Mood and anxiety questionnaires annoy me.”
- “I can accurately score my pain experience with 1 to 10 numbers.”
- “To keep track of psychosocial factors is important in understanding my pain.”
- “To keep track of environmental factors like weather or pollution, is important in understanding my pain.”
- “I prefer to use humor to describe my pain experience.”
- “I prefer to use drama and story-telling to describe my pain experience.”
- “I prefer scientific terminology to describe my pain experience.”

These questions were developed as a result of feedback received during our two-day public drop-in event for PwPPs, during which we explored some of the challenges of documenting CP experience and creative ways to facilitate communication of pain as a qualitative and quantitative experience (59, 60).

### Statistical analysis and reporting

All survey responses are presented as descriptive statistics (counts or percentage of part/group responses, plotted with bar and Likert charts, respectively. We used SPSS V.27 for MAC. Multivariable questions about the ICT repertoire under study are shown as radar plots of the response ratios, to provide a multidimensional overview of between-group and between-system differences in risk assessments.

All comments were reviewed, and if any provided additional information or insight they were included within Results.

## Results

### Participant characteristics

Of 202 survey responses received by June 2021, 128 identified as female, 32 as male; 0 as other; and 42 did not answer the question of gender. The sample was comprised of persons with persistent pain (PwPP) (100 female, 23 male, 4 not answered), caregivers (18 female, 4 male), Medical professionals such as nurses, doctors or healthcare workers (17 female, 4 male, 25 not answered) and others who were interested but were not in any of those categories (27 female, 8 male). Fifty respondents had indicted more than one reason for joining the study. For example, 20/46 Medical professionals were also PwPP; or 14/22 caregivers were also PwPP, etc. For this reason, we identified mutually exclusive groups based on first who was a PwPP (*n* = *127*); next by who was a Medical professional but not PwPP (*n* = *26*); the rest (who were neither Medical professionals nor PwPP) were classified under Other (*n* = *49*).

The reported average age of PwPPs (43.86 ± 14.97) was close to the rest (42.11 ± 17.38). Nearly one third of PwPPs earned an income < $25,000 (CAD$). Fewer than 27% of PwPPs worked full-time; 9.8% were unemployed, 13% on disability leave, 10.6% retired, 13% students, and 13% indicated other situations (specified as maternity leave, student while working, retired and still working, self-employed, etc.).

### Characteristics of PwPP and their coping strategies

Primary and secondary chronic pain were reported by our respondents. The most prevalent condition was Lower back pain (*n* = 72), followed by Headache (*n* = 58), Arthritis (*n* = 35), Fibromyalgia (*n* = 33), neurogenic pain (*n* = 28); post-trauma (*n* = 22), post-surgical (*n* = 20), unknown (*n* = 18), abdominal (*n* = 18); musculoskeletal (*n* = 11), Cancer (*n* = 4), endometriosis (*n* = 3), and ehlers-danlos syndrome (*n* = 1).

**Figure 1:**
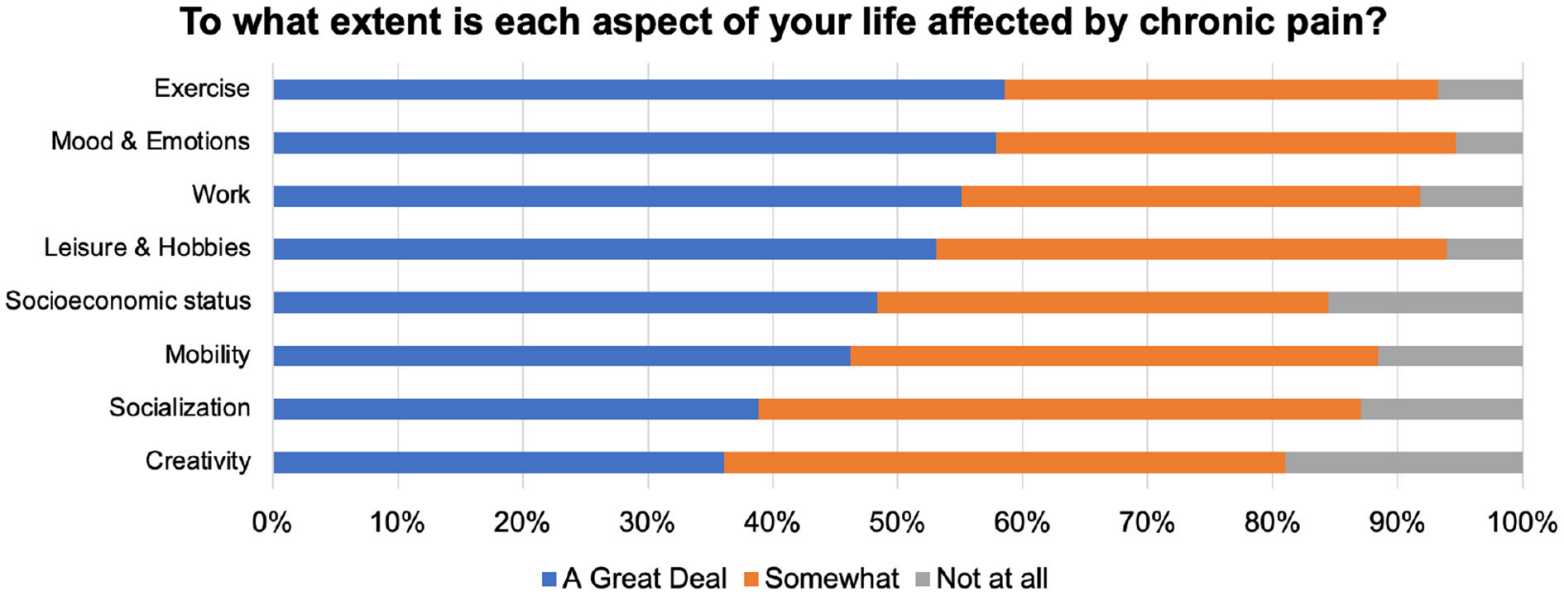
Impact of CP on different aspects of life (*n* = 127).

All aspects of life (primarily, exercise and physical activity) were impacted as a result of chronic pain (Figure 1). Consistently, among the various coping strategies that we listed, and those suggested by respondents in their comments, alternative physical therapies such as massage (*n* = 47*)*, acupuncture (*n* = 17), homeopathy (*n* = 8), and chiropractic (*n* = *6)* were cumulatively more frequently mentioned.

**Figure 2:**
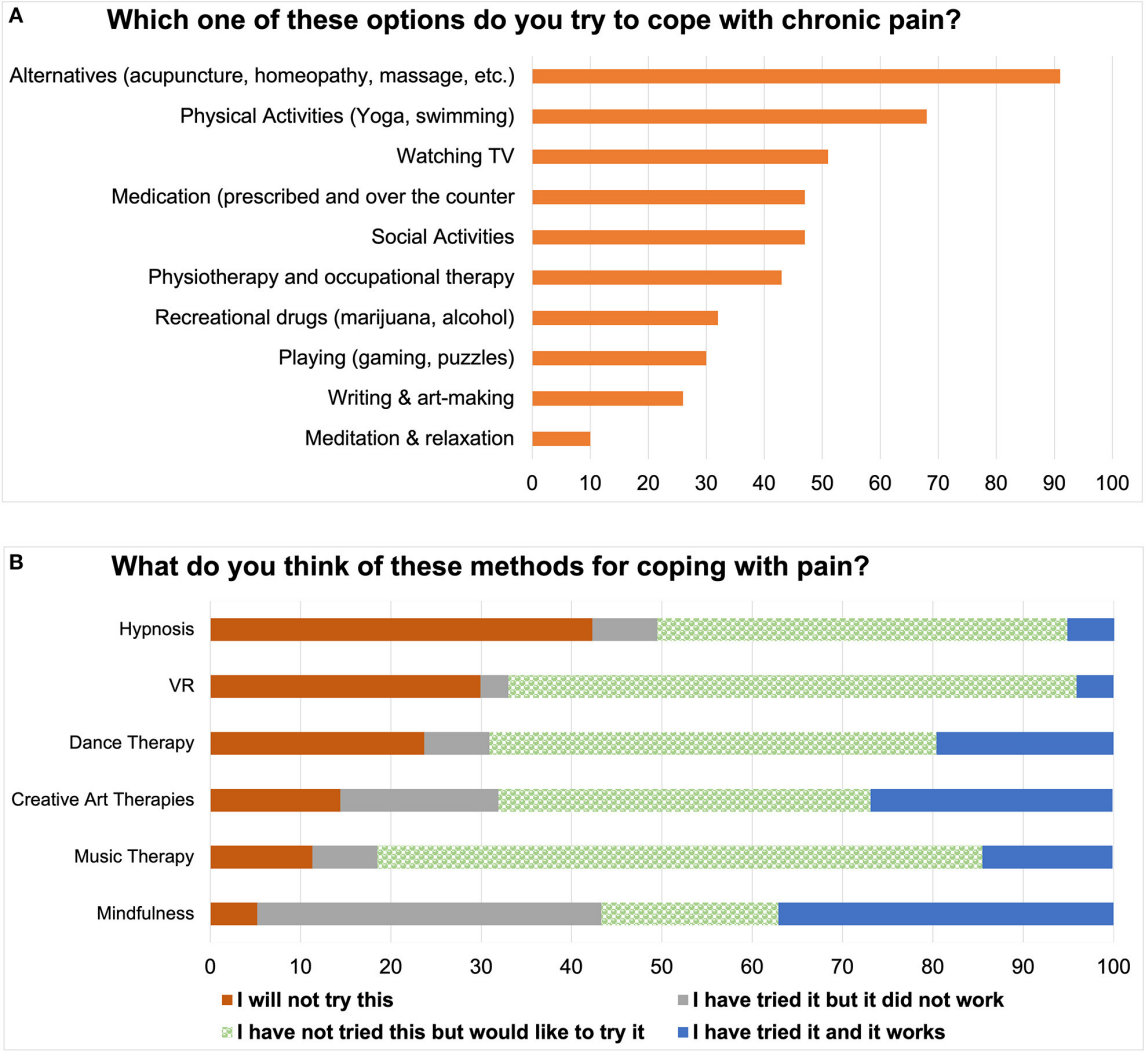
Coping strategies among PwPPs. **(A)** Common strategies; **(B)** Newer strategies.

We listed a number of possible activities that PwPPs might use for pain coping, and asked them to add any that were missing. As can be seen in Figure 2A, various forms of complementary and alternative therapies were mentioned. We asked about attitudes towards several other alternative therapies that may be offered to PwPPs (Figure 2B) and found that in general there was a positive attitude towards trying interventions that were new to the respondents. Figure 2B also reveals that asking questions about a general method (e.g., mindfulness) may not be sufficient to capture nuances of the technique used. Here, it can be seen that although nearly 70% of respondents had experience with mindfulness, it was effective in only half of them.

### Attitudes towards alternative medicine

**Figure 3:**
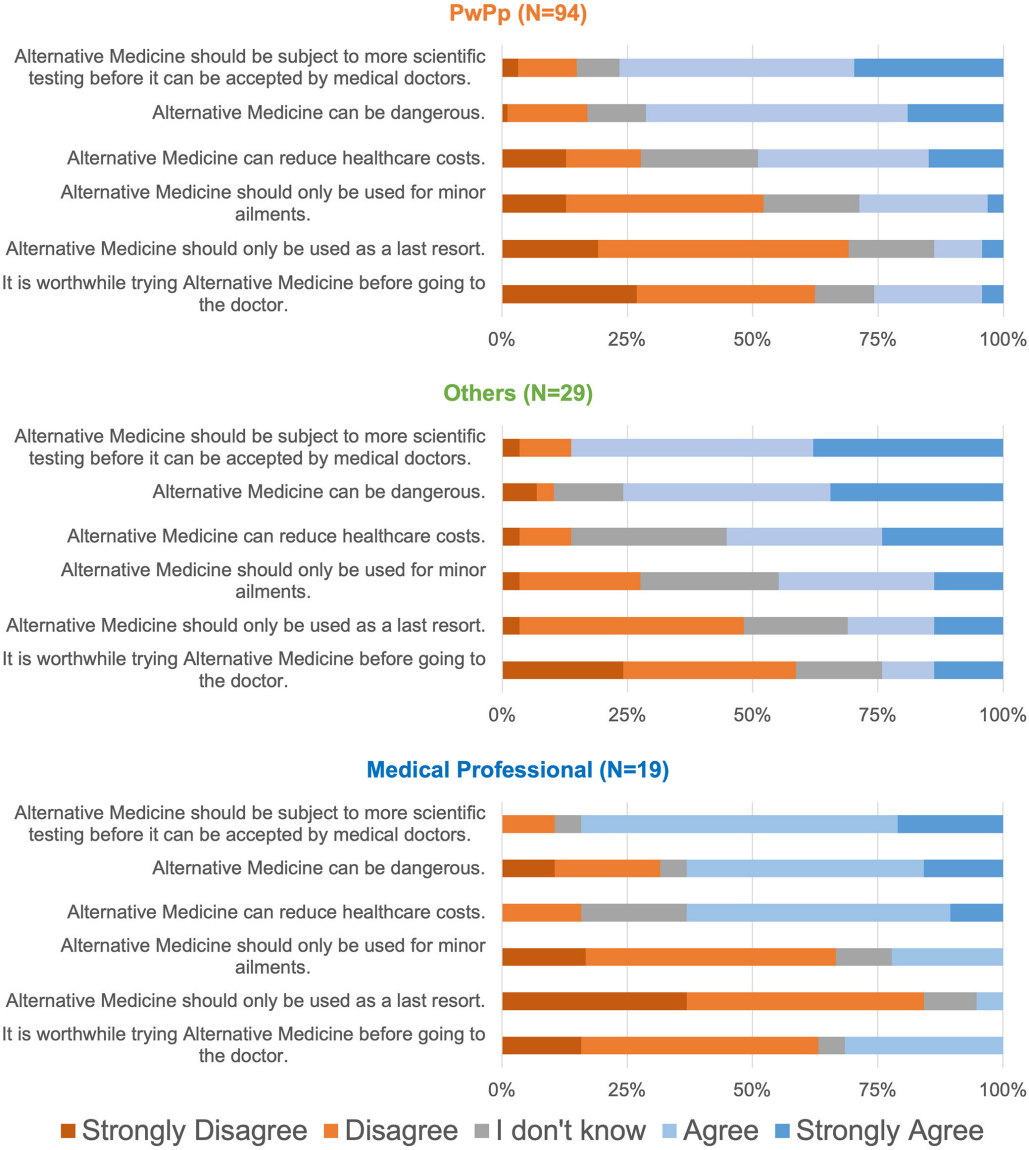
Attitudes towards alternative medicine.

Only 142/202 respondents offered comments on questions about attitudes towards alternative medicine. As Figure 3 illustrates, more than 75% of all three groups agreed that there is a need for more medical testing of alternative medicine to be accepted by doctors. Proportionately, fewer respondents believed it to be dangerous and more than 25% (including medical professionals) agreed that it was worthwhile trying it before seeing a doctors. Interestingly, more than 50% of respondents, especially medical professionals *Agreed* or *Strongly Agreed* that alternative medicine can reduce healthcare costs. We interpret these results as an indication of ambivalence and hope that further research in alternative medicine can produce beneficial results. The Kruskal-Wallis test did not reveal significant between-group differences but in two questions (that alternative medicine should be used for minor ailments (*p* = 0.014), and that alternative Medicine should be only used as a last resort (*p* = 0.002); with the Other group expressing a more positive attitude compared to medical professionals). Post-hoc comparison of attitudes among medical professionals who were also PwPPs *vs*. the rest did not reveal any differences.

### General attitude towards using ICTs to cope with illness

**Figure 4:**
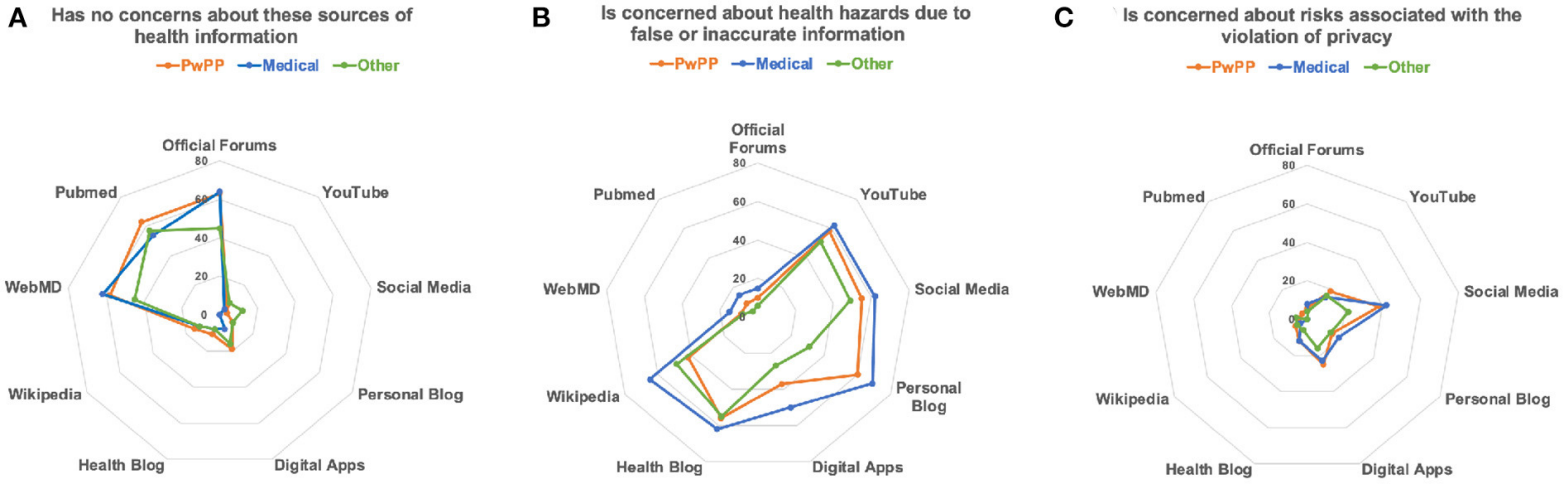
These radar diagrams illustrate differences in attitudes towards possible digital health information systems: **(A)** No concern; **(B)** Risks of wrong information; **(C)** Risk of violation of privacy. The radius of each point corresponds to the percentage of an affirmative responses within each group.

Considering the affordances of coping with illness and pain digitally (9), we envisioned various types of ICTs that could facilitate knowledge generation and dissemination in a citizen science framework. As Figure 4 illustrates, professionally-maintained websites such as WebMD, PubMed and official forums were seen as the most trustworthy health information forums. Respondents did, however, express moderate concern about risks associated with flawed or incomplete information being communicated *via* YouTube, social media, blogs, and Wikipedia. Interestingly, there was significantly less concern about potential privacy violations on social media and digital apps than about hazards of inaccurate information. As can be seen from the radar plots in Figure 4, the responses of PwPPs and Medical professionals were remarkably similar (with medical professionals being overall more cautious). The Others were comparatively less concerned about safety hazards and privacy issues.

### Familiarity with mHealth

**Figure 5:**
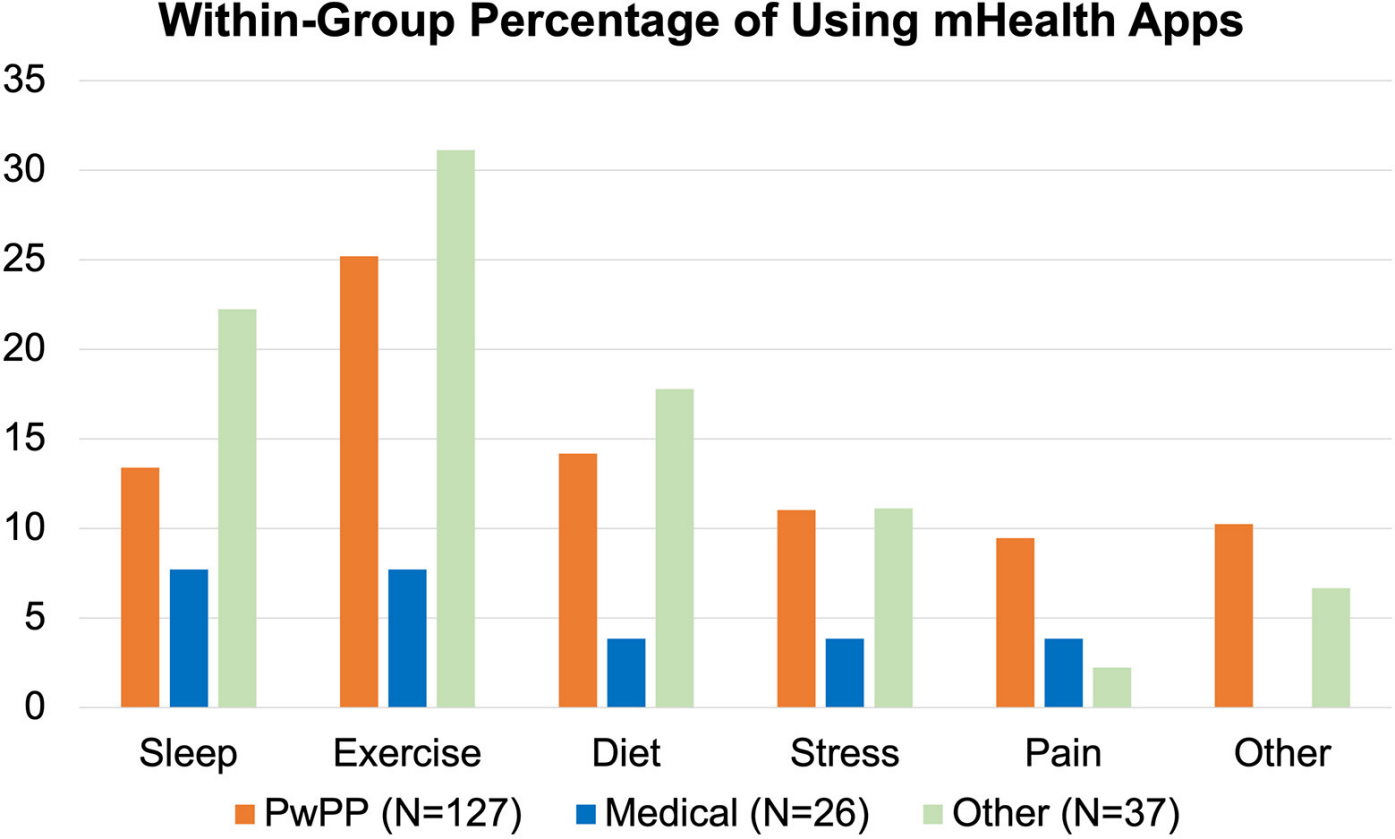
Percentage of mHealth App users within each respondent group. Medical and Other groups shown here are mutually exclusive from the PwPP category.

Despite high access to smartphones (92%) and tablets (67%) (which was comparable to Others and lower than Medical professionals), only 53% of PwPPs (*n* = 57) were familiar with digital self-tracking apps. As shown in Figure 5, Medical professionals were the least likely to be using mHealth apps. Compared to Others, the rate of mHealth app usage was lower among PwPPs and only a small number were familiar with pain tracking apps. In addition to the functions listed in Figure 5, respondents added apps for tracking; menstrual cycle (*n* = 6), weight-watching (*n* = 2), heart monitoring (*n* = 2), and mindfulness (*n* = 2).

Familiarity with pain tracking systems was also low. In response to “*Have you ever used a pain tracking and pain management app or computer program?”*, <20% (*n* = 21*)* of PwPPs responded “Yes.” When asked about the frequency with which such systems were used, few indicated the use of pain tracking when pain symptoms flared up (*n* = 4) or to track physical activity (*n* = 2).

Neither access to technology nor digital literacy could explain these differences. Among the 107 PwPPs who replied to the question “*Please tell us how often you use a tablet or phone for each of the following activities?”* 98% indicated email and text messaging; 95% indicated surfing the net; 79% for pictures and videos; 77% for social media; 75% for news media; 45% for self-tracking apps; and 29% for playing digital games. Kruskal Wallis tests did not reveal any statistically significant difference between PwPPs, Medical professionals and Others in terms of their usage.

### Appraisal of apps for self-tracking and management

**Figure 6:**
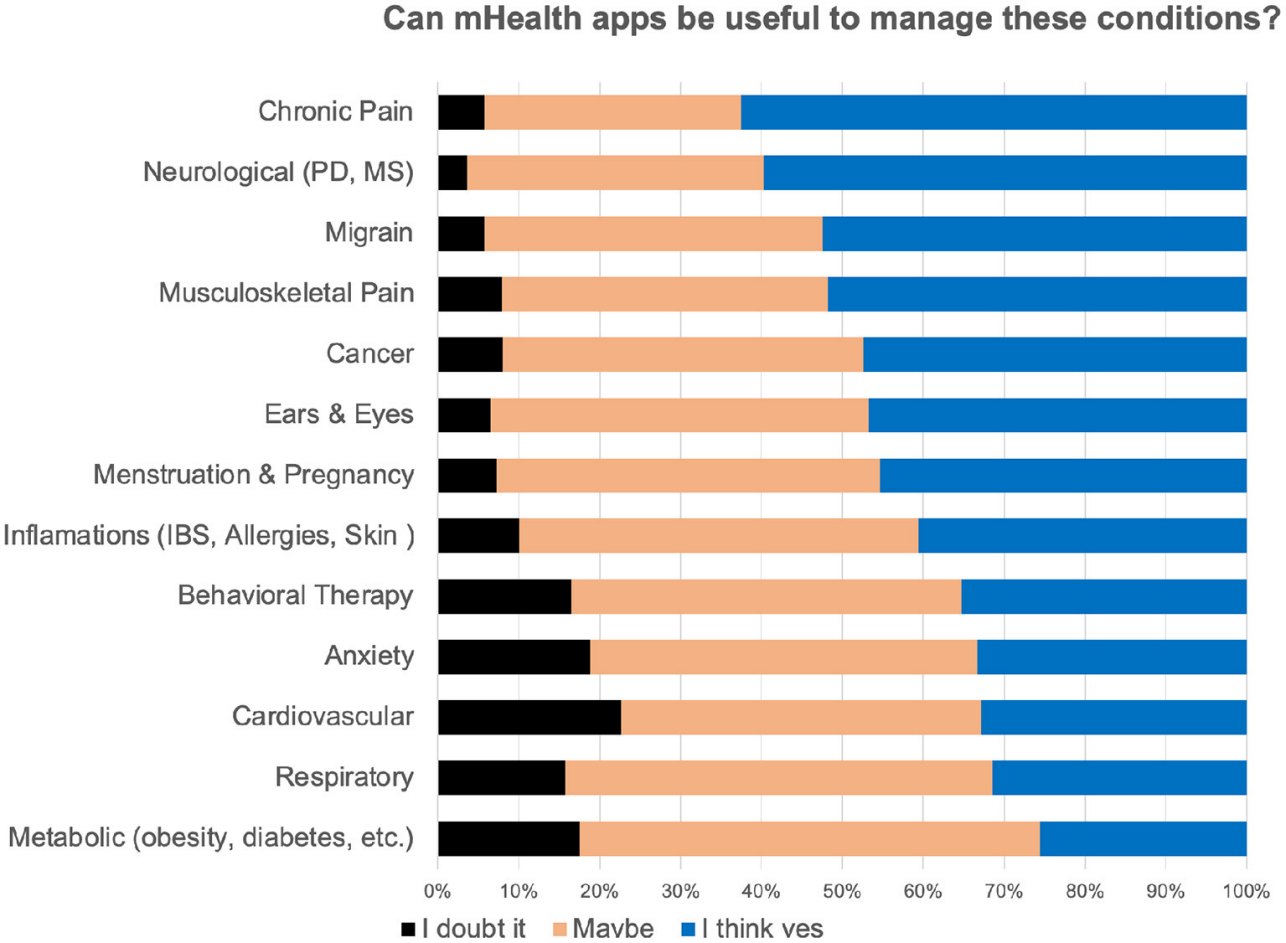
Beliefs about the usefulness of mHealth tracking apps for different chronic conditions.

Attitudes towards digital self-management of chronic conditions were generally positive. In response to the query “*Do you think apps can be useful to manage any of the following CHRONIC HEALTH CONDITIONS?”*, the majority (62%) selected the response: 'I think yes' for pain management. As can be seen in Figure 6, although certainty about effectiveness of such systems varied across conditions, the ratios of “I doubt it” responses were minimal across all chronic conditions. One surprising finding was that, similarly to cardiovascular and metabolic disorders, anxiety was not perceived as a condition to benefit from such systems.

### Appraisal of apps for data generation and sharing

General attitudes towards data sharing *via* apps were positive. More than 72% of respondents answered “Yes” to the question “*Would you be open to using a health-related app as a research tool for data collection in real life situations?”*. Less than 2% responded “No,” while 25.4% responded “Maybe.” When asked: “*Do you believe that by using apps to collect your own data, you can help advance pain research?”*, 35% responded “Definitely yes”, and 53% responded “Possibly.” Less than 10% responded “I don't know”, with only 3 responding “I don't think so.” In response to the question: “*Would you be comfortable sharing your data collected in an app anonymously for specific health research purposes?”* 75% responded “Yes;” 13% replied “Maybe;” and only 7% selected “No.”

**Figure 7:**
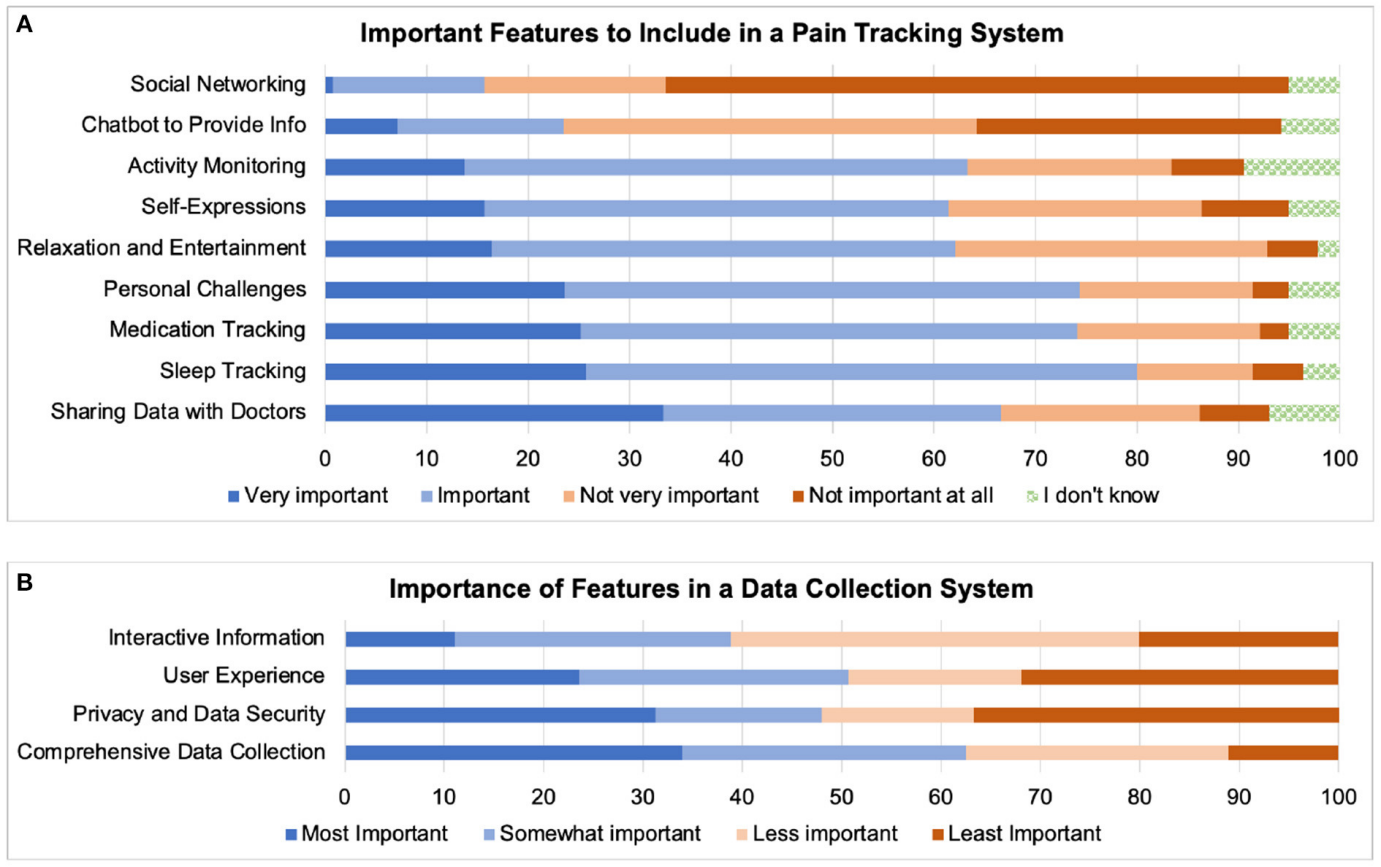
Desired features for **(A)** Pain tracking; **(B)** Data sharing.

We listed features common to self-tracking apps, and asked respondents to rate the importance of these (Figure 7A). The ability to share data with their doctors was the most important feature, followed by tracking of sleep. Those familiar with pain tracking apps also mentioned additional features such as receiving reminders (*n* = 1) or tips (*n* = 2), personalized predictive clues (*n* = 5), visual cues for pain recording (*n* = 1), and relaxing and pleasant experience (*n* = 3). When we asked respondents to rank the importance of features in a digital data collection system, comprehensive data collection scored the highest (Figure 7B).

### Reflective analysis of comments

Excluding comments that offered specifics about type of pain and work status, we reviewed all comments about the usefulness of apps (19), data collection (11), improvement to features (23), reasons for using apps (11), concerns (6), and how best to express pain (12). We list themes that emerged from comments (all made by respondents who identified as female), that better illustrate which factors may motivate participation in our digital citizen lab, and which may contribute to technostress.

#### Positive appraisal 1: Contributing complex data

Commenting on the question of what motivated them to join the study, a 64-year old woman with lower back pain explained:

*I developed chronic pain only about 2 years ago and have done a lot of personal research about how to reduce the pain through diet and exercise. I feel that I have hit a plateau and am looking at other alternative ways of discovering more about pain and its causes*.

In further elaborating on the question of the type of pain, she elaborated:

*I cannot classify with absolute certainty the reason or source of the pain. I have been to see my doctor and had extensive blood work but nothing is conclusive. Other than the pain, especially in my lower back, neck and hips I am in very good health. I do not know if the pain is Psychogenic*.

This individual had access to Samsung Health app and monitored her exercise level but was not familiar enough with other features of the apps to answer the question: “how do you feel about existing apps in the market?”

*It is hard for me to answer the questions as I have very little experience with health apps. I am however looking forward to participating fully in this research and learn to properly use your health app*.

In terms of data collection, she believed that tracking psychosocial factors, as well as environmental factors such as weather and pollutions was very important for understanding her pain, and was willing to contribute pain-tracking data (quantitative, narrative, expressive, psychometric):

*Overall I am open to trying any of the above mentioned methods of pain tracking for my own benefit and the benefit of the research*.

She had a wide range of activities in which she engaged to cope with her pain, including:

*Journaling, researching and using nutrition to discover pain sources, exchanging information with a group of friends who are as informed and curious as me, sharing my information and comparing with this group of friends, looking for alternatives, yoga, meditation*.

In explaining her response to the question: *Do you believe that by using apps to collect your own data, you can help advance pain research?*, she wrote:

*I chose “possibly” because I do not want to have unrealistic expectations*.

#### Positive appraisal 2: Data aggregation towards personalization of care

Reviewing the 23 comments in response to “*What would you like to improve in your existing self-tracking apps?”* revealed interest in 'smarter apps' capable of data aggregation and algorithmic personalization of tips offered to users suffering complex neurogenic pains such as Fibromyalgia.

For example, a 53-year-old woman with neurogenic pain (rating her general health as very bad) who used apps for monitoring her irregular heartbeat wished for:

“*Input for triggers and resolution tactics. What caused the pain and what helped mediate it.”*

This respondent relied on meditation, massage therapy and recreational drugs, as well as art and literature to cope with her general poor health conditions.

One 34-year-old woman with Fibromyalgia, who rated her general health as very bad, and used a pain tracking app daily, wished for:

“*aggregating data and making better correlations over time. For instance, my app says I feel more pain on slower days but doesn't see that I'm resting more because I have more pain. If I rest more one day and feel better the next it doesn't track that.”*

A third woman (51-year-old) with Fibromyalgia and an unknown type of pain persisting after an accident (rating her General Health as 'okay'), with daily user of pain tracking apps wished for additional features:

“tips or links to resources for point-of-need techniques such as meditation, soothing sounds, breathing exercises etc.”

This respondent believed that the data collection could be successful depending on:

“*how well planned and constructed the app is, and how rigorously analysed the resulting data is.”*

#### Negative appraisal 1: Difficulty of access and use

Several comments of those who were familiar with mHealth indicated dissatisfaction with interfaces:

“*I have not tried a great many, but I am easily overwhelmed by them.”*

The added burden of technology was noted:

“*It's hard to update an app when your [sic] in dying in pain”*.

The numerousness of apps that have not been satisfactory, and their costs, were also mentioned:

“*I have tried many apps for period tracking, exercise tracing, and sleep tracking, and I have found them all disappointing or annoying in some way. I don't know how to choose. I also don't want to pay monthly membership fees, but it's frustrating that the highest rated apps are all paywalled and the free ones are full of ads or not very good.”*

#### Negative appraisal 2: Futility

To perceive a new technology as futile is an impediment to its exploration and adoption. We noted two types of negative appraisals, related to personal beneficence, and related to beliefs about feasibility.

An important example of negative appraisal was offered by a 36-year-old professional woman with facial chronic pain, who did not use any mHealth applications. She indicated that although she had created her own self-tracking computer program, she had stopped using it:

“*I am not sure if it is a good thing to track pain. I stopped doing it because looking back over the months and years I could see that I had rarely had a pain free day, and that was very discouraging.”* This respondent preferred to not focus on data-collection: “*One of the best strategies for me is to ignore it and get on with things. Thinking about it seems to make it worse.”* She also felt that when one lives with untreated pain for a long time, then tracking it will not serve any purpose: “ *I don't see the value* [of using apps] *in my own case because my issues is still not really diagnosed properly there doesn't seem to be much value in tracking it after already having one so for quite a while*.”

Others cast doubt on the feasibility of making sense of, or analyzing, the type of data that could be collected in our proposed scheme.

In response to “Do you believe that by using apps to collect your own data, you can help advance pain research?” one 34-year-old woman (identified as an unemployed PwPP, without access to a smartphone or tablet) responded “I don't think so” and elaborated further:

“*Chronic pain is so broad in how it is experienced. Unless the data collected is for a very specific experience/condition, I think it's likely just adding to the mountain of info that nobody seems to know how to climb.”*

This respondent was one of ten (out of 123) who did not believe that they could accurately score their pain experience with 1–10 numbers. She was also among the minority of respondents who were annoyed by mood and anxiety questionnaires. She somewhat agreed about the importance of psychosocial factors, scientific terminology and use of humor to describe her pain experience. Instead, she considered tracking environmental factors as very important to understanding her pain (headache, lower back pain and joint pain). Nevertheless, in response to “*Would you be open to using a health-related app as a research tool for data collection in a real life situation*?,” she replied “Maybe.”

Concern about the validity of data generation was shared by a 61-year-old woman with a PhD in nursing. Similarly to the comments of the 34-year-old woman above, she did not believe that she could accurately score her pain experience with numbers. Although she was not a user of any mHealth apps, and was open to using them for health research, she thought that the possibility of the research succeeding depended on:

“*whether the data collected and the intervention provided actually target* [one's] *chronic pain*.”

#### Negative appraisal 3: Ambiguity and intersubjectivity of pain

**Figure 8:**
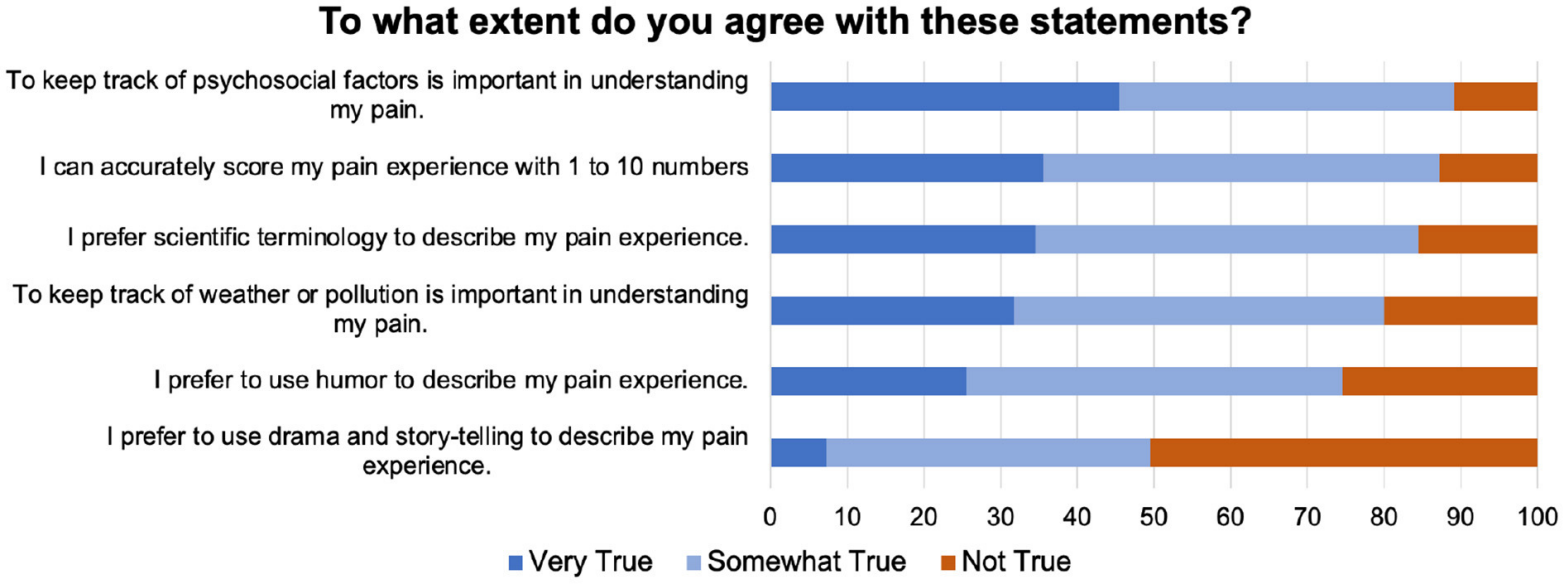
Questions asked about different ways of providing pain-related data to solicit comments from participants.

As Figure 8shows, when asked to evaluate statements about how to communicate their pain experience, a large majority was interested in psychosocial determinants of pain and more open to using scientific terminology or psychometric questionnaires.

Additional comments revealed a concern with data interpretation:

A 46-year-old woman who identified as PwPP who did not use any mHealth apps, commented on the transactional nature of pain communication:

“* The description of pain and how a person feels changes with who they are interacting with. Some people may only be comfortable with humour for example so I stick with that when talking to them. Talking about chronic pain makes many people uncomfortable because they don't know how to react/what to say in response.”*

Although this individual was not a user of mHealth apps, and strongly agreed with the statement:

“*Health apps in the market are not properly tested by medical professionals.”*

She believed that mHealth apps could help with all chronic conditions listed in the survey, and strongly agreed that by sharing her data she could advance pain research by helping others understand her better:

“*I have Psoriatic Arthritis. My condition has been dismissed (to my face) by health professionals who do not understand my condition and pain levels. I think that advancements in pain research and management are very much needed. Pain is very complex and makes every aspect of life difficult- something most people rarely think about.”*

Interestingly, a retired 78-year-old woman who identified as artist and poet (did not own a smartphone or tablet, and was not familiar with mHealth apps) commented on the novelty of both health apps and pain communication. In response to “Is there anything that you think would improve your pain tracking app?” she commented:

“*I never knew there was such an app. I'd be tempted to buy a cell phone to try it out. Or maybe an Apple watch? Or maybe some other appliance?”*

When asked about different ways to capture one's pain experience, she commented:

“*Truthfully, I never thought about expressing my pain to anybody much since it wears on anyone's patience to be overexposed to complaints.”*

A 77-year-old female retired nurse who also identified as PwPP (owned both a smartphone and tablet, but did not use any mHealth apps) and preferred scientific terminology for description of her pain, commented that:

“*I can find it difficult to quantify situations that are complex in a way that will fit a questionnaire.”*

## Discussion

### Summary of findings

Within the theoretical framework of stress research (in recognition of the fact that innovative proposals such as DiSPORA—aiming to facilitate patient-partnership in research—may cause technostress), this quasi-qualitative attitudinal user study assessed the affordances of mHealth applications to create a digital citizen laboratory for person-centered research of alternative (non medical) pain management.

As expected, CP affected various aspects of PwPP's lives (Figure 1). The majority of PwPPs resorted to various alternative therapies such as acupuncture, massage, chiropractic, homeopathy mode than medications (Figure 2A). The majority of PwPPs in this study were also open to exploring more recent modalities (VR, mindfulness, art therapy), although efficacy of treatment (e.g., in mindfulness) varied among those who had tried them (Figure 2B). The majority of respondents (PwPPs, medical professionals and the rest) required more research to make alternative medicine acceptable to doctors. While the majority agreed that alternative medicine could be dangerous, nearly half of the respondents (especially 12/19 medical professionals who were not PwPPs) believed that alternative medicine could be saving healthcare costs (Figure 3).

Given the fact that since 2015 nearly 300 pain apps have been launched for personal and research purposes (19), we were surprised that despite having access to smartphones and tablet, and being digitally literate, only a small percentage of PwPPs were using such apps. Nevertheless, overall attitudes toward possible benefits of these apps were positive—especially for pain management.

Despite their novelty, more than 88% of respondents to our survey considered data collection through mHealth apps as *possibly* (53%) or *definitely beneficial* (35%). More than 70% of PwPPs were willing to participate in digital pain research, and were comfortable with sharing their anonymized data collected in an app. Although privacy was one of the more important requirements, the need for comprehensive data collection was ranked higher (Figure 7).

Reflective analysis of comments revealed three themes: (1) Positive appraisals were related to the affordances of mHealth apps for collecting complex personal data, and data aggregation towards personalized pain treatments. (2) Negative appraisals were about the futility of data collection about a condition that could not be cured and futility of collecting data that could not be meaningfully interpreted. (3)Technostress was associated with costs of acquiring such technologies, under experience, and understanding of the inner workings of the app and privacy.

### New contributions to qualitative pain research

#### A framed-flexible approach

To the best of our knowledge, this is the first study to have approached the question of creating an mHealth app for citizen research into alternative means of coping with CP. In the first step of the recursive testing of appraisal, we chose an anonymous survey to provide an opportunity for candid appraisals. By inviting individuals to share ideas and knowledge for the creation of a hypothetical data-collection app aimed to break the typical hierarchy of subject-matter experts and participants, and sought to create a playful and welcoming atmosphere to encourage free self-expression.

To create a citizen lab for studying coping necessitates identifying users' activities (e.g., pain management strategies), motivations for engagement, and technological skills and attitudes (62, 63), as well as accounting for the fact that patients communicate their pain creatively (64–67). Addressing all of these factors in detail will have created an extensive list of questions that would normally be explored in a quantitative manner. However, an attitudinal research survey that was informed by conversations among our team (researchers, PwPPs and caregivers) helped us begin from a low-resolution but wide angled viewpoint that was informed by our team's own ambivalences: Is it *safe* and *acceptable* to create a mobile self-tracking app for communicating and documenting alternative self-care? Informed by previous user-centered studies (65–72), we asked questions about features that are known to be important to pain-tracking app users: ability to track pain accurately, to interact, to provide descriptive information about pain, and finally to distract from pain. This allowed us to ask whether there were concerns about these well-desired features (Figure 7).

One of the respondent's comments on the question listing different means of explaining their pain experience (scientific, humor, numeric scales, etc.) exemplifies the types of information that our study design sought to gather: Who has difficulty with expressing their chronic pain, and how do they cope with it?

“*I've really never thought my own pain was severe enough to warrant attention or tracking. It exists, but it's not debilitating the vast majority of the time. I also think I have a fairly high pain tolerance—which is to say, I don't really know what objective number I would assign to pain because I feel able to handle it, even if it's severe. Also, less severe/sharp pain (such as my shoulder) is sometimes harder for me to cope with than a severe, acute pain. So, while I would objectively say that delivering a baby at home with no pain meds is very painful (I have done it three times), the shoulder pain is harder to cope with psychologically. I keep coming back to the shoulder because it's the least explained pain I've had for the longest period of time.”*

We found that the strongest desire for data collection was expressed for sleep-tracking (something that should be done at the comfort of home and passively), and for sharing personal data with their doctors (something that requires active interactions and knowledge exchange). The fact that collecting comprehensive data was more important than ensuring privacy suggests the willingness of potential participants to be agents in capturing the complexity of their pain experiences, particularly psychosocial factors (Figure 8).

#### Barriers to digital citizen labs

Respondents to our call were interested and curious about alternative ways of coping with pain (Figure 2), even when acknowledging potential dangers and the fact that standard medical care should be the primary resource, and the need for more research and evidence (Figure 3). Nevertheless, more than half of participants, especially Medical professionals believed that alternative medicine could potentially save healthcare costs. This is consistent with the WHO report, that interest in traditional and complementary medicine is globally on the rise, but that resources and legal and ethical frameworks for research and implementation are lagging (41).

In addition, a lack of established health ICT frameworks and clear policies contributes to negative appraisals and technostress. It is not surprising that our participants found misinformation, privacy issues, and skepticism about interfaces, costs and algorithms to be areas of concern (73, 74).

To remedy, based on a scoping review of digital health science initiatives, Fu, Gray and Borda have suggested that participatory design of research data management systems would be an important step in overcoming hesitancy about the reliability and reproducibility of citizen health data collection initiatives (75). Hamilton et al.'s patient engagement research has identified that procedural requirements which ensure that data collection is consistent with the needs of both research and patients—and make it possible to do research at their own pace and with the ability to express their own views—are important to the design of citizen research practices (76).

Thomas, Scheller, and Schroder have recommended that an effective citizen laboratory for addressing complex questions (in our case, coping with chronic pain) must provide (1) a space for social encounters; (2) a framework for communicative practice; (3) a process to initiate social self-understanding; (4) and dynamics to engage in (counter-)public discourses (77). Our data suggest a foreseeable challenge in gaining trust about communication. As can be seen in Figure 7A, inclusion of a social networking feature in a pain tracking system was not at all important to the majority of respondents, and social media raised concerns about misinformation (Figure 4). It should be noted however, that our questions were too broad to tease apart the perceived benefits or risks of personal connections *via* ICTs and this issue needs to be more thoroughly examined in the future.

#### Beyond quantitative scales and towards innovative data collection

In a critical review of existing citizen science methodologies and approaches, Hidalgo et al. (78) have identified two dominant forms of citizen science. In the contributory model, scientists are the project designers and offer a technology to help citizens gather their data. In the co-creative and participatory model, citizens share their real-world problems and scientists are co-designers and facilitators of data tools that emerge from the real-world problems of citizen participants. There is a wealth of existing data on what users expect from pain-tracking apps, designed for therapeutic efficacy (64, 68–72). Our data suggests that the contributory model of citizen science is acceptable to our respondents. However, important work remains to be done to raise awareness about the critical role that patients can play in guiding the direction of research through communication of personal and shared experiences and complex needs (78).

An area that requires more precise research is alternative methods to pain communications (going beyond numeric pain scales). We had expected that, given the complexity of pain experience, self-expressive and metaphoric pain communication be important (64–67, 79). We found that although facilitating self-expression was just as important as physical activity tracking (Figure 7A), only half of participants were interested in performative approaches such as drama and storytelling (narrative reporting) for reporting their pain (Figure 8). We speculate that this might be due to negative connotations associated with the word “drama,” that reflect the pervasive stigma of malingering (80) for women in particular, who represent the majority of our respondents. However, as several comments indicated, how one expresses and describes their pain is contextual and dependent on to whom the explanation is addressed. Therefore, while explaining pain in scientific terms or with numerical scales may simplify the challenges of intersubjectivity, welcoming alternative means of self-expression in pain research may be informative.

As one respondent commented:

“*because I am not a scientist, I don't know how to express my pain in scientific terms*.”

Additionally some were not comfortable 'burdening' others with their pain complaints. Future work should more closely examine the affordances of poetic and metaphorical self-expression in a digital citizen lab.

### Limitations

The most important limitation of this study is the potential for sampling bias: our sampling method provided for self-selection (based on interest in joining a future participatory app design study). Thus, the respondents are not representative of the PwPP population. This self-selection resulted in a group of more than 73% female, educated, mostly English-speaking individuals (even though the study was also advertised in French) with high access to ICT. In reality, many PwPPs are in marginalized communities (2) without access to ICTs, and our study may not have reached them. For example, a recent rapid review of studies that examine the efficacy of mHealth qualities illustrates a great disparity in accounting for preferences of mHealth use in lower income countries (81).

Another potential bias is that most respondents reported lower back pain. Some estimates of the lifetime prevalence of chronic back pain “are as high as 84% in the adult population” (82). This disproportionate representation in our sample might be related to the fact that almost 60% (75/127) learned about our study from the PERFORM Centre's mailing list and had likely been involved in the other studies for treatment of the lower back pain (for which physical therapies exist) as opposed to migraine, for example. Future research should target a more diverse range of chronic pain conditions.

In this study, we chose not to employ any formally validated questionnaires, and instead allowed conversations with PwPPs (in an earlier public event) to guide the formulation of questions. As such, we can only provide descriptive statistics, with limited quantitative comparison across the sample. Recently published scales such as The Digital Stressor Instrument would be useful in conjunction with our survey, to obtain a more granular understanding of the ways in which digital stress may impact PwPPs (51).

## Conclusion

This attitudinal user study showed that our survey respondents were interested in alternative therapies, and willing to share complex personal data, to advance pain research through data-analytics. The key finding of our study is that despite novelty and uncertainty about the outcomes of innovative health related ICT approaches, those with CP conditions and the medical professionals are open to researching CP in non-pharmacological and alternative frameworks. Although the primary appraisal of a digital citizen lab for exploring alternative ways of coping with and studying chronic pain is positive, heterogeneity in both positive and negative attitudes must be more carefully studied, and individual perspectives and experiences be considered in designing digital frameworks for citizen research. Specifically, respondents who commented about the study expressed doubts that computational algorithms would be successful in making meaningful inferences from the data, because pain is a complex experience and difficult to communicate and record. In future work, we need to reach those who did not have an opportunity or chose not to participate in our call. While a more representative sample, and more in-depth engagement is needed to plan DiSPORA, current results underline the necessity of clinically-framed, but flexibly person-centered and psychosocially-informed research and medical care for PwPPs.

## Data availability statement

The dataset may contain personal and identifiable information requests to access the datasets should be directed to najmeh.khalili-mahani@concordia.ca.

## Ethics statement

The studies involving human participants were reviewed and approved by Concordia University's Research Ethics Board. The patients/participants provided their written informed consent to participate in this study.

## Author contributions

NK-M, SW, EH, AP, and MR: designed the study. NK-M, EH, and AP: conducted data collection and analysis. All authors contributed to the writing of the manuscript.

## Funding

The funding for this study was provided by AUDACE-2019 grant from Fonds de recherche du Québec - Société et culture (FRQSC) to NK-M and MR.

## Conflict of interest

The authors declare that the research was conducted in the absence of any commercial or financial relationships that could be construed as a potential conflict of interest.

## Publisher's note

All claims expressed in this article are solely those of the authors and do not necessarily represent those of their affiliated organizations, or those of the publisher, the editors and the reviewers. Any product that may be evaluated in this article, or claim that may be made by its manufacturer, is not guaranteed or endorsed by the publisher.
